# Histone glycation: Linking metabolic perturbation with epigenetic misregulation in cancer

**DOI:** 10.3934/genet.2019.2.14

**Published:** 2019-06-04

**Authors:** Xiayao Diao

**Affiliations:** Department of Urology, Sun Yat-sen Memorial Hospital, Sun Yat-sen University, 33 Yingfeng Road, Haizhu District, Guangzhou 510220, P. R. China

Unlike healthy cells, tumor cells have their own unique metabolic features [1]. Emerging evidence in recent years indicates that dysregulation of metabolism allows tumor cells to modulate epigenetic marks to support tumor initiation and progression [2]. Therefore, how tumor-induced metabolic changes alter the epigenetic landscape, especially modifications on histones, thereby promoting malignant transformation, adaptation to inadequate nutrition and metastasis has become a hotspot. Tumor cells primarily rely on anaerobic glycolysis for energy production (Warburg effect) [1]. This metabolic feature resulting in high levels of reactive oxygen species (ROS) and reactive carbohydrate species such as methylglyoxal (MGO). Indeed, MGO accumulation was identified in many types of cancers [3].

Condensation of MGO with reactive amino acid residues via the Maillard reaction thereby forming stable adducts is a major characteristic of glycation [4]. The initial glycation adduct can further oxidize to form a series of stable products, which can undergo additional chemical transformations including the ability to form cross-links, yielding species generally referred to as advanced glycation end-products (AGEs) [4].

The core histone protein (H2A, H2B, H3 and H4), which spool eukaryotic DNA into a chromatin structure, contains an unstructured N-terminal tail that extends away from the nucleosome core particle (NCP). It undergoes a variety of post-translational modifications (PTMs), including methylation, acetylation and ubiquitination by a range of chromatin effectors that can read, write and erase these modifications [5]. Histone PTMs play a crucial role in determining cell fate by establishing and maintaining the epigenetic landscape [6]. A recent analysis of histone glycation has shown that several sites on linker histone H1 and H2B were found to be modified with various AGEs, including sites known to carry enzymatically added PTMs [7]. However, little is known about histone glycation in cancer and how it affects chromatin structure and biological behavior of cancer cells.

In a study recently published in Nature Communicatios, titled “Reversible histone glycation is associated with disease-related changes in chromatin architecture”, Zheng et al. [8] performed a thorough analysis of the occurrence mechanistic effect and pathological implications of histone MGO glycation in tumor cells and uncovered a novel molecular mechanism linking metabolic perturbation with epigenetic misregulation in cancer.

In this study, the authors conducted a comprehensive analysis at both low and high resolutions utilizing a variety of methods examining the occurrence as well as the local and global chromatin changes induced by histone glycation in physiological and breast cancer samples. Since MGO is an important glycolysis by-product, they applied a range of MGO concentrations corresponding to different sites: MGO ratios to test the reactivity of MGO and chromatin components in vitro. They found that chromatinized histone MGO glycation disrupts global histone enzymatic PTM levels. H3R8me2 showed higher sensitive to MGO treatment than lysine methylation of H3K4 and H3K9, which corresponds with the higher reactivity of MGO towards arginine over lysine.

To test whether MGO adducts could have a significant effect on nucleosome stability, the authors performed a “one-pot” nucleosome assembly in the presence of unmodified or glycated histones. The results indicate that high concentrations of MGO can induce both histone-histone and histone-DNA cross-linking in chromatin, potentially having harmful effects on its dynamic nature.

Furthermore, they utilized reconstituted nucleosomal arrays composed of 12 repeats of 601 DNA assembled in a similar manner to NCPs and found that glycation-induced DNA-histone cross-linking on nucleosomes can affect the architecture of chromatin on both a local and global scale.

DJ-1 has emerged in recent years as a protector of metabolism-associated cellular stress, reversing primarily ROS damage in neurons but also protein and DNA glycation under a variety of conditions [9]. In addition, DJ-1 was defined as an oncogene due to its overexpression in many cancers, as well as the fact that its knockdown decreases cancer cell proliferation and induces their apoptosis [10]. In this study, the authors synthesized a peptide corresponding to the H3 N-terminal tail with a C-terminal biotin and subjected it to MGO treatment followed by incubation with recombinant DJ-1 and streptavidin pull down. The results indicated that DJ-1 is a potential regulator of histone glycation.

Finally, the authors examined histone glycation and sensitivity to MGO in breast cancer cells because breast cancer was shown to have a particularly high metabolic rate and ROS levels. Their results indicated that all tested cell lines have both higher basal H3 glycation as well as sensitivity to MGO treatment. Consistence with the data observed in cell culture, analyzing histones extracted from breast cancer xenograft tumors revealed most of them contain high basal histone MGO glycation levels. Interestingly, the expression level of DJ-1 was positively correlated with the histone MGO glycation levels in cell lines and xenograft tumors.

Taken together, these findings reveal the pathophysiological accumulation of histone glycation and identify an additional molecular mechanism linking metabolic perturbation with epigenetic misregulation in cancer. This study uncovered a novel mechanism for cellular metabolic damage through epigenetic perturbation and proposed at therapeutic avenue, targeting DJ-1 as a key gatekeeper of cancer cell survival.
